# *ClickX*: a visualization-based program for preprocessing of serial crystallography data

**DOI:** 10.1107/S1600576719005363

**Published:** 2019-05-28

**Authors:** Xuanxuan Li, Chufeng Li, Haiguang Liu

**Affiliations:** a Department of Engineering Physics, Tsinghua University, Beijing 100084, People’s Republic of China; b Complex Systems Division, Beijing Computational Science Research Center, ZPark II, Haidian, Beijing 100193, People’s Republic of China; c Department of Physics, Arizona State University, Tempe, AZ 85287, USA

**Keywords:** serial crystallography, X-ray free-electron lasers, XFELs, data visualization, data analysis, Python

## Abstract

Serial crystallography is a powerful technique in structure determination using many small crystals at X-ray free-electron laser or synchrotron radiation facilities. The large diffraction data volumes require high-throughput software to preprocess the raw images for subsequent analysis. *ClickX* is a program designated for serial crystallography data preprocessing, capable of rapid data sorting for online feedback and peak-finding refinement by parameter optimization. The graphical user interface (GUI) provides convenient access to various operations such as pattern visualization, statistics plotting and parameter tuning. A batch job module is implemented to facilitate large-data-volume processing. A two-step geometry calibration for single-panel detectors is also integrated into the GUI, where the beam center and detector tilting angles are optimized using an ellipse center shifting method first, then all six parameters, including the photon energy and detector distance, are refined together using a residual minimization method. Implemented in Python, *ClickX* has good portability and extensibility, so that it can be installed, configured and used on any computing platform that provides a Python interface or common data file format. *ClickX* has been tested in online analysis at the Pohang Accelerator Laboratory X-ray Free-Electron Laser, Korea, and the Linac Coherent Light Source, USA. It has also been applied in post-experimental data analysis. The source code is available via https://github.com/LiuLab-CSRC/ClickX under a GNU General Public License.

## Abstract 2

A Python-based program for serial crystallography experimental data preprocessing is developed for both online and offline analysis. Enhanced features include a graphical user interface, batch job execution and fast parameter optimizations.

## 1. Introduction   

In recent years, great success has been achieved in macromolecular structure determination by serial femtosecond crystallography (SFX) (Chapman *et al.*, 2011 ▸; Boutet *et al.*, 2012 ▸; Barends *et al.*, 2014 ▸). Using extremely bright X-ray free-electron laser (XFEL) pulses, a diffraction signal can be detected to atomic resolution with micrometre-sized crystals at room temperature under a ‘diffract-before-destroy’ scheme (Neutze *et al.*, 2000 ▸). This allows structure determination in ambient environments, thus significantly reducing structural alteration during the cryo-cooling process (Fraser *et al.*, 2011 ▸; Keedy *et al.*, 2014 ▸) adopted in diffraction experiments using synchrotron light sources. The femtosecond duration of XFEL pulses provides the unique advantage of probing fast dynamics in pump–probe experiments, particularly useful in understanding light-triggered processes (Kupitz *et al.*, 2014 ▸; Tenboer *et al.*, 2014 ▸; Pande *et al.*, 2016 ▸; Nogly *et al.*, 2018 ▸). If femto­second temporal resolution is not desired, the idea of SFX can be extended to the serial crystallography (SX) at synchrotrons with microfocus crystallography beamlines (Nogly *et al.*, 2015 ▸).

In typical SX (including SFX) experiments, millions of raw images are collected to yield a complete data set that can be used for high-resolution structure determination (Liu & Spence, 2016 ▸). Furthermore, these data are often collected at high repetition rates, generating high throughput of raw data for the downstream analysis. For example, the Coherent X-ray Imaging instrument (CXI) (Liang *et al.*, 2015 ▸) at the Linac Coherent Light Source (LCLS), USA, allows diffraction data to be collected at a repetition rate of up to 120 Hz. However, only a small fraction of the raw data contains useful diffraction signals that can be processed to a merged 3D diffraction intensity map for final structure determination. Data reduction is the first step of the SX data analysis pipeline, where raw data frames with a number of diffraction peaks are identified as valid diffraction data (hits) for further analysis, such as indexing, scaling and merging. High-throughput programs with fast processing capability are required to provide real-time feedback to guide experiments. Furthermore, the indexing rate of SX diffraction data is sensitively dependent on the number of Bragg peaks. To reduce the inclusion of artifacts recognized as peaks (false positives) and exclusion of actual peaks (false negatives), several rounds of parameter fine tuning are necessary to yield optimal outcomes.

Currently there are several programs for SX data preprocessing. *CASS* (Foucar, 2016 ▸) is a modular program that can be used in both single-particle and SFX experiments. *Cheetah* (Barty *et al.*, 2014 ▸) provides rapid data reduction and a convenient graphical interface, *Cheetah-GUI*, for batch job management. Python-based *cctbx.xfel* (Sauter *et al.*, 2013 ▸) provides an alternative data analysis pipeline, including preprocessing (*e.g.* geometry optimization and spot finding) and post-processing (*e.g.* indexing and merging). *IOTA* (Lyubimov *et al.*, 2016 ▸) is a submodule of *cctbx.xfel* to optimize spot-finding parameters using a grid-search method. *OnDA* (Mariani *et al.*, 2016 ▸) is an online monitoring program that offers fast feedback on hit rates since it reads detector data directly from memory at LCLS. *NanoPeakCell* (Coquelle *et al.*, 2015 ▸) and *Psocake* (Thayer *et al.*, 2016 ▸; Damiani *et al.*, 2016 ▸) are Python-based graphical user interface (GUI) programs which provide data visualization and preprocessing support.

In this article, we introduce a software package for SX data analysis, with a focus on raw data preprocessing: *ClickX. ClickX* is implemented in Python, and therefore the program is easy to read and update and is highly portable for various platforms. *ClickX* is organized in modules, with the following major features:

(1) Cross platform, easy installation. As a Python program, *ClickX* can be downloaded and immediately run on any platform with Python environments.

(2) Fast speed and high parallelization. *ClickX* extensively utilizes numerical packages including *NumPy* (https://www.numpy.org/) and *SciPy* (Jones *et al.*, 2001 ▸), so the processing speed is comparable to C/C++ software, such as *Cheetah*. Parallel computing is implemented with *mpi4py* (https://mpi4py.readthedocs.io/), gaining a linear speedup if input/output (I/O) is not a limiting factor.

(3) Real-time parameter tuning and feedback. *ClickX* provides two hit finders (SNR model and Poisson model, see Section 2.4 for details) and a GUI for real-time parameter tuning. Hit-finding results and statistics will be displayed immediately following the change of parameters.

(4) Highly automated data processing. A job management system is implemented for job submission and execution, and the program takes care of the data analysis in batches with a single click.

(5) Geometry calibration. *ClickX* integrates a calibration module to optimize geometry parameters such as the beam center, detector tilting angles, detector distance and photon energy.

(6) Data visualization. *ClickX* supports multiple data formats, and users can visualize a broad range of data types, including pixel values, individual diffraction patterns and statistical data from analyses.

## 2. Functions and implementations   

### 2.1. Data visualization   

**Figure 1:**
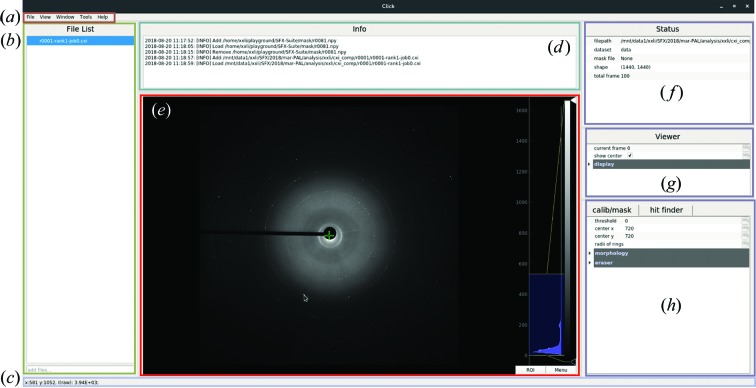
Layout overview of the main interface. (*a*) Menu bar. All other modules and widgets (*e.g.* batch module and geometry calibration module) can be accessed here. (*b*) File list panel. Data files can be added by dragging and dropping for further use. (*c*) Status bar. The pixel value and coordinates are displayed for inspection. (*d*) Info panel. Important events and messages are shown here. (*e*) Image viewer. The green ‘+’ symbol shows the beam center. Users can zoom in/out or drag the displayed image for detailed checking. The intensity histogram is shown on the right-hand side, where the color bar can be altered via mouse dragging. (*f*) Status panel. Information on the current displayed file and mask file is shown here. (*g*) Viewer panel. Users can jump to a specified frame in the current data set. (*h*) Parameter panel. Users can create a mask and tune hit-finding parameters.

In data processing of SX experiments, visualization is helpful for users to get a comprehensive understanding of data, tune parameters and diagnose problems. *ClickX* provides a user-friendly interface (Fig. 1 ▸) to work with data stored in various formats, including npy, npz, HDF5 and CXI (Maia, 2012 ▸). Most operations can be performed with a mouse (point–click–drag), including adding/removing files, selecting and loading data sets, zooming in/out on images, tuning the color bar, and inspecting pixel values.

### 2.2. Basic analysis of diffraction images   

**Figure 2:**
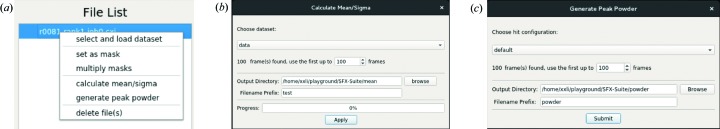
Basic analysis. (*a*) Right-click file items in the file list panel. Users can perform some basic analyses. (*b*) Mean/sigma image generation dialog. (*c*) Peak powder generation dialog.

Some basic statistical analyses (Fig. 2 ▸) are implemented in *ClickX*, including the following:

(*a*) Generating the average intensity image and the associated variance image, *i.e.* mean/sigma value of each pixel. The average and standard deviation images can be generated from designated data sets, which can be used for mask preparation.

(*b*) Generating a powder diffraction image from hits. A powder diffraction pattern can be generated on the basis of the peaks identified with a user-specified hit-finding configuration. This can be used to calibrate experimental geometry using the calibration module to optimize the parameters associated with the detector.

(*c*) Composing masks. Users can combine multiple masks to generate a composed mask for more specific hit-finding tasks.

### 2.3. Mask building   

**Figure 3:**
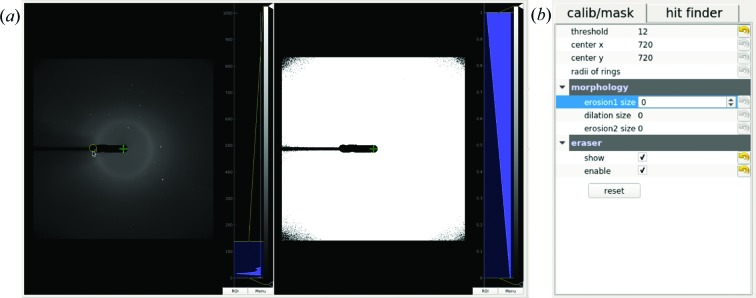
Mask building. (*a*) Raw image viewer (left) and calib/mask viewer (right). The yellow circle under the cursor is the mask eraser. Users can change the size and move around to create a custom mask. The generated mask is applied to the raw image viewer immediately and is also shown in the calib/mask viewer. (*b*) Calib/mask tab in the parameter panel.

During data collection of SX experiments, some pixels can be dysfunctional for various reasons, such as being shadowed by beam stops, intensity saturation, overheating *etc*. These pixels should be masked out for subsequent analysis. Fig. 3 ▸ shows mask-related widgets, where the mean/sigma images are used as primary references to build a mask. A threshold is applied to generate a binary mask, which can be further refined by morphology operations (dilation and erosion). *ClickX* also supports a flexible mask generation tool, *mask eraser*, which can be used to generate an arbitrary mask by dragging and erasing operations within the GUI. This feature facilitates the creation of masks composed of regions with arbitrary shapes.

### 2.4. Hit finding   

Hit finding is the most critical step in preprocessing of SX data, and it is also the core function in *ClickX*. We implemented two hit finders: the SNR (signal-to-noise ratio) model and the Poisson model (Lan *et al.*, 2018 ▸). The former approach evaluates peaks on the basis of SNR values, which is similar to the zaef method implemented in *CrystFEL* (White *et al.*, 2012 ▸), while the latter considers the statistical significance under the assumption of a Poisson distribution.

#### 2.4.1. SNR model   

In the SNR model, the raw image is first smoothed by gaussian_filter, a Gaussian filter function implemented in *SciPy* (Jones *et al.*, 2001 ▸), to suppress background noise. The smoothed image is then used to calculate a gradient image for initial peak identification. Raw peaks in valid regions of gradient images are found by a local maximum searching method, which is implemented in the *scikit-image* (van der Walt *et al.*, 2014 ▸) package. All raw peaks are then evaluated on the basis of the SNR values. The number of signal pixels will be examined to screen spurious peaks resulting from sources other than crystals.

The SNR is estimated in a 7 × 7 cropped region centered on each peak candidate: 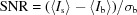, where 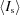 is the mean value of the signal region, and 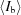 and 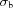 are the mean and standard derivation values of background region. The signal and background regions can be determined using three methods:

(1) Rings method. This method is similar to the ring integration method in *CrystFEL*. Each peak is described using three concentric circles, whose radius parameters are specified to define the signal and background regions. The smallest radius defines the signal region and other two radii define an annulus background region.

(2) Simple method. The signal and background regions are defined on the basis of the intensity values. By default, the 10 pixels (∼20% of pixels in the cropped 7 × 7 image) with the highest intensities are selected as signal pixels, while the 35 pixels with the lowest intensities are treated as background pixels.

(3) Adaptive method. The lowest 70% of pixels (the percentage can be specified by the user) form the background region. The signal threshold is calculated from the background region: 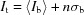, where *n* is a user-specified parameter which is set to 5 by default.

#### 2.4.2. Poisson model   

The Poisson model assumes that the photon counts follow Poisson distributions. For a pixel with a background value *b*, the probability of obtaining a photon count *K* is 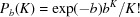. We can define a photon threshold 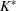 by the cumulative probability
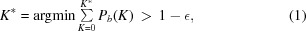
where ∊ is set to a very small number, *e.g.* 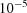. A pixel is set as a signal pixel if its photon count is higher than the threshold 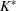. Regions with connected signal pixels will be considered as peak candidates.

In the Poisson model, the raw image is first converted to a photon image by dividing ADU (analog–digital units) by the number of photons. A threshold image is then calculated on the basis of the Poisson distribution, where the background value is calculated by averaging photon counts in pixels located in the same ring. Initial peak candidates are identified from connected regions of the photon image with higher values than the threshold image. They are further filtered by the mask and the number of signal pixels, which can be adjusted by users.

In practice, the SNR-adaptive method is recommended in the first round of analysis. The detector layout (for multi-panel detectors such as the CSPAD at LCLS) and ADU per photon are required for hit finding using the Poisson model. The SNR-rings and SNR-simple methods have constraints on the sizes of the signal and background regions, determined by the user-specified parameters. The SNR-adaptive method only has one parameter controlling the size of the background region, and the signal region is determined by calculated the threshold from the background region and a user-specified parameter *n*. This will give more flexibility in signal pixel detection, which is advantageous in extracting information from peaks with large size variations.

#### 2.4.3. Performance test   

Speed tests of *ClickX* were carried out on a computer with a six-core Intel Xeon E5-2603 @ 1.7 GHz. A data set composed of 1000 frames, each with 1440 × 1440 pixels, was processed using the SNR-adaptive method three times on between one and five workers (one CPU core per worker). The result shows that the processing speed of *ClickX* scales linearly with the number of workers. On average, 3.5 frames per second per core can be achieved, so ∼35 (34 workers + 1 master) cores are required to process raw images at 120 Hz if I/O is not the limiting factor.

A comparison between *ClickX* and *Cheetah* has also been conducted. According to the analysis of the data sets recently collected at the Pohang Accelerator Laboratory X-ray Free-Electron Laser (PAL-XFEL), Korea, the hit-finding results are improved compared with those from *Cheetah*. However, we cannot conclude that *ClickX* outperforms *Cheetah*, since the hit-finding results depend on the parameter setup and characteristics of experimental samples. The main advantage is that *ClickX* gives user controls immediately accessible from the GUI and fast feedback so that it is easier to obtain optimal hit-finding parameters.

### 2.5. Parameter tuning   

**Figure 4:**
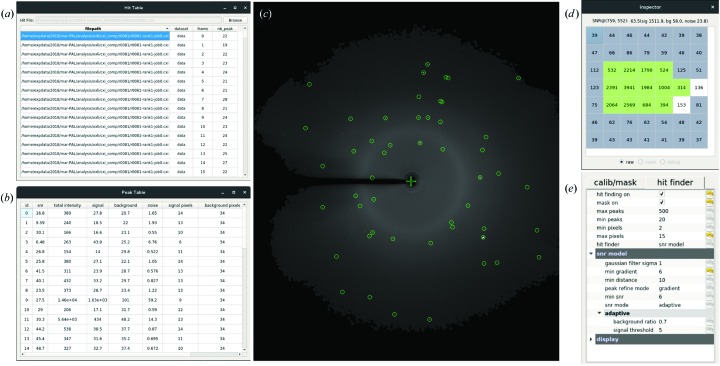
Parameter-tuning widgets. (*a*) Hit table. Hit-finding job results such as file path, frame and number of found peaks are shown in this table. Users can sort the table by clicking the corresponding header such as nb_peak. By double-clicking any row, the user can jump to the corresponding frame in the main interface for further inspection. (*b*) Peak table. Information on identified peaks of the current image is shown in this sortable table. If the user double-clicks any row, the image viewer of the main interface will zoom in on the corresponding peak. (*c*) Image viewer with identified peaks shown in green circles. (*d*) Pixel inspector. A crop under the cursor of the current image is shown, including signal pixels (in green boxes) and background ones (in gray boxes) with intensity values. Statistics such as SNR, signal, background and noise are shown at the top of the panel. (*e*) Hit finder tab in the parameter panel. All changes of parameters will take effect immediately and found peaks will be plotted on the central image viewer.

In *ClickX*, multiple widgets are implemented for parameter tuning (Fig. 4 ▸). Users can change parameters within the GUI in real time without the need to edit and specify a new configuration file or interrupt the ongoing process. Identified peaks are highlighted on the current image. Statistics such as SNR, total intensity and number of signal pixels are available in a peak table. This fast feedback can help users with hit-finding-parameter fine tuning. Once the hit-finding parameters are satisfactory, users can submit batch jobs to process/reprocess all data sets.

### 2.6. Geometry calibration   

An accurate geometry is important for indexing. Because XFEL experiments often require fast readout, multi-panel detectors, such as the CSPAD, are used to match the data collection rate. The relative placements of each panel should be optimized to achieve accurate indexing results. Such functions have been reported to be available in *cctbx.xfel* (Hattne *et al.*, 2014 ▸), *geoptimiser* (Yefanov *et al.*, 2015 ▸) in *CrystFEL*, *DIALS* (Waterman *et al.*, 2016 ▸; Brewster *et al.*, 2018 ▸) and *cppxfel* (Ginn & Stuart, 2017 ▸). In *ClickX*, we focus on refining parameters associated with single-panel detectors (or multi-panel detectors with well determined metrology) using powder diffraction signals, including the photon energy (λ), detector distance (*D*), beam center (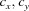) and detector tilting angles (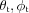). The multi-panel detectors require more information than powder diffraction data to optimize the geometry for each panel independently. It is noted that hit finding is a local operation, so it does not rely on accurate metrology or geometry information.

**Figure 5:**
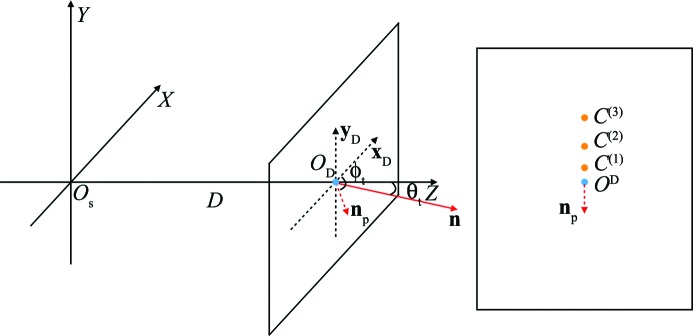
Geometry setup. The centers of powder rings 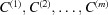 are used to optimize the beam center and detector tilting angles in the first step.

Fig. 5 ▸ shows the geometry setup in *ClickX*. 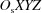 is the Cartesian coordinate system of SX experiments, where 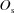 is the scattering point and *Z* is the beam direction. 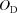 is the beam center on the detector. *D* is the distance between the detector and the crystal sample. 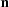 is the normal vector of the detector, which is parallel with the *Z* direction if the detector is not tilted. 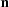 can be represented by two tilting angles: polar angle 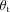 and azimuthal angle 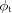. 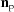 is the projected vector of axis *Z* on the detector plane. 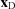/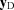 is the projected vector of the *X*/*Y* axis on the detector. 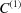, 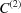,…, 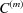 are the centers of powder rings.

The calibration process consists of two steps. The first step optimizes the beam center and detector tilting angles using a small-tilting approximation method. It utilizes the fact that the powder diffraction rays form a series of concentric conics which intersect with the detector, resulting in circular rings that share the same center if the incident beam is perpendicular to the detector (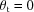). If tilting exists, the rings become elliptical and the centers are not overlapped. The centers of these ellipses are located on the same line, which is parallel with 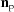.

With some approximations (see detailed derivation in Appendix *A* [App appa]), we can prove that
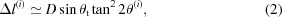
where 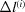 is the distance between the beam center and the center of the powder ring *i* at scattering angle 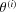 (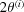 is the angle between the incident beam and scattered beam directions of ring *i*).

The first step is implemented as follows:

(1) Clustering. In calibration experiments, usually inorganic crystals with small unit cells are used to collect powder patterns. This avoids the complexity of powder ring overlapping. Given *N* powder peaks from *m* powder rings, the radius of each peak is calculated, and then the DBSCAN method (Ester *et al.*, 1996 ▸) is used to group peaks into *m* clusters. Each ring forms a cluster, so peaks in this cluster share the same *d* spacing of the calibrant. Each cluster is evaluated to obtain its number of peaks 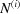, center coordinate 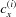, 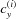, and scattering angle 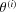.

(2) 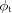 estimation. A linear regression is performed on the cluster centers, and 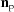 can be calculated from the regression results:
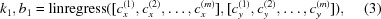


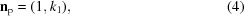
where *k*_1_ and *b*_1_ represent the slope and intercept of this linear regression, respectively. The azimuthal angle 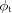 is the angle between 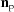 and 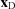.

(3) 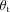 estimation. For ring *i*, 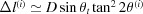, and then the distance between the center of ring *i* and the first ring is 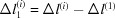. A linear regression is performed on a series of 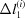 and 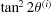:
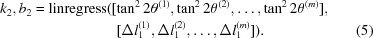
The polar tilting angle 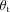 can be estimated using 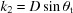.

(4) Beam-center estimation. In the previous step, we have 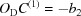, so the coordinate of the beam center 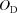 can be determined from 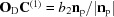.

(5) Optimization evaluation. To evaluate the goodness of fit after the optimization, the residual between the calculated 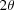 and its model value of powder peaks can be calculated by
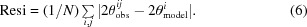


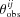
 is the scattering angle of peak *j* on ring *i* based on current geometry parameters, while 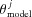 is the model value of the scattering angle of ring *i*. In step (1), this model value is the mean value of all calculated scattering angles on this ring.

After the first step of optimization, a further refinement can be performed by minimizing the residual in the second step, which is similar to the approach taken in *FIT2D* (Hammersley *et al.*, 1996 ▸; Hammersley, 2016 ▸). The model value in this step is calculated from the photon energy and *d* spacings of the calibrant. The Nelder–Mead (Gao & Han, 2012 ▸) method is used to minimize the residual to obtain the final geometry parameters.

**Figure 6:**
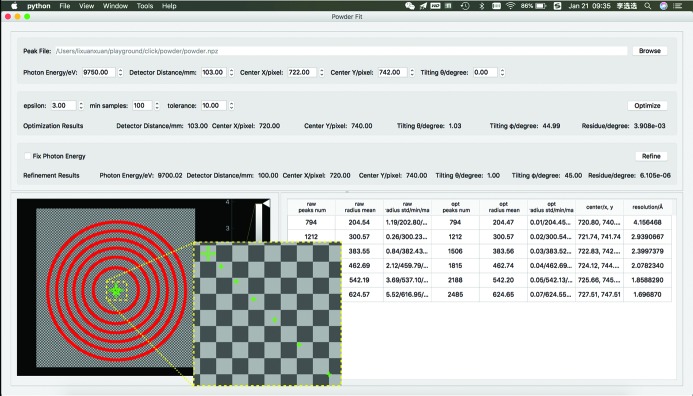
Geometry calibration module. Peaks shown in red are clustered into multiple rings. The centers of rings are shown as small green ‘+’ symbols, while the fitted beam center is shown as a large green ‘+’ symbol. Detailed results of clustering and fitting are shown in the bottom-right table.

Fig. 6 ▸ shows a screenshot of a geometry calibration. For geometry calibration, a peak powder file is required as input. The powder image can be generated using the powder generator in the main interface of the *ClickX* program. Given appropriate initial geometry parameters, users can perform two-step calibration quickly.

### 2.7. Batch job system   

**Figure 7:**
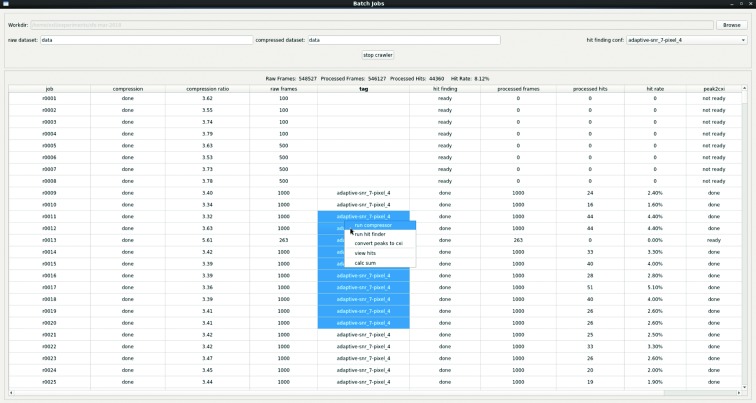
Batch job system. Detailed results of jobs are shown in the job table. Users can right-click the table to submit corresponding jobs.

In SX experiments, terabytes of data are collected and organized in tens, even hundreds, of data files to reduce the burden of I/O. To analyze such large data sets efficiently, a batch job system (Fig. 7 ▸) was implemented. Users can submit jobs and check the status and statistics of each job. Furthermore, an auto-processing mode can be enabled, so that jobs will be managed by the batch job system.

Currently *ClickX* supports three functions in batch mode:

(1) Data compression. Raw data can be compressed into CXI files with the HDF5 compression technique, which saves a significant amount of storage if users need to save or transport raw data sets.

(2) Hit finding. Users can execute hit finding with specified configurations obtained from the main interface.

(3) Peak2CXI conversion. If the hit-finding results are satisfactory, users can save image frames identified as hits and associated peak information in CXI files for further analysis; these files follow the format defined by the Coherent X-ray Imaging Data Bank (Maia, 2012 ▸).

Some useful statistics are summarized in the main panel of the batch job window, including job progress, number of raw frames, number of processed frames, number of hits, hit rate *etc*. After hit finding, users can visually inspect the identified hits in the main interface. On the basis of the outcomes, users can take appropriate actions, such as refining parameters for another round of hit-finding analysis.

#### 2.7.1. Parallel implementation   

**Figure 8:**
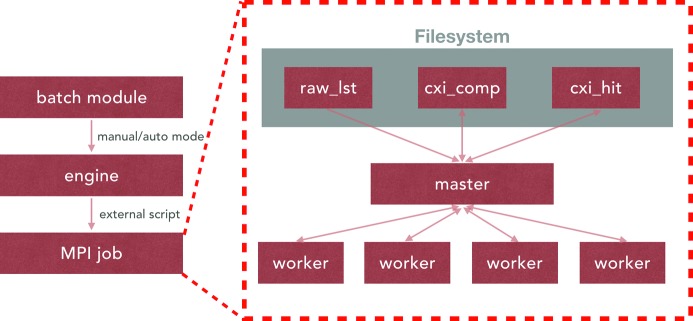
Parallel implementation. The engine accepts jobs from the batch module, and they are executed in a master/worker architecture. The master node collects job information from specified folders and dispatches jobs to workers dynamically. The results are sent to the master and saved in corresponding folders.

The batch job system is implemented in an efficient and extensible manner (Fig. 8 ▸). To ensure that the batch system can be used on any platform, an interface layer is implemented as the parallel engine. The available engines are for either local machines or PBS-configured clusters. The engines can be expanded to include other types of parallel systems, such as IBM’s LSF or SLURM. Jobs can be submitted to the engine manually or automatically when the data are ready. The engine will run corresponding MPI jobs with external scripts.

The GUI of *ClickX* communicates with jobs via buffering files. All analysis projects using *ClickX* follow the same format for data and outputs. The raw data files to be processed are compiled to a list file and saved to the raw_lst folder. Compressed data are saved in the cxi_comp folder. Hit-finding results are saved in the cxi_hit folder.

All jobs are executed in the master/worker paradigm. When a new job is sent to the engine, the master will first collect job information from the corresponding folders or files, then split the multi-frame job into many single-frame job batches, and dispatch them to workers dynamically to balance the loads at the worker. The master is responsible for collecting analysis results from workers and saving them to corresponding folders.

## 3. Workflow with *ClickX*   

**Figure 9:**
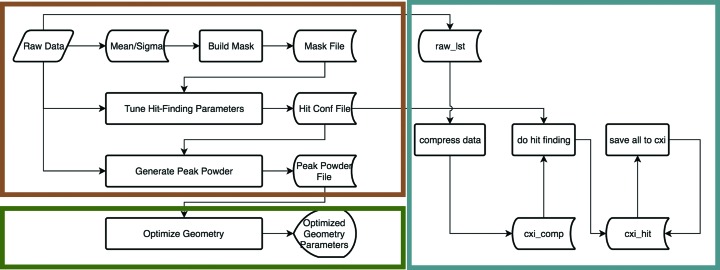
Workflow with *ClickX*. *ClickX* is organized in three modules: the main interface (top left), the geometry calibration module (bottom left) and the batch job system (right). Data compression is optional in the batch system. Hit finding can be performed directly on the raw data in raw_lst if compression is not required.

As a modular program, *ClickX* is designed to provide users with full control of SX data preprocessing via three independent but also interconnected modules. Fig. 9 ▸ shows a basic workflow to process SX data with *ClickX*. In the main interface, preparation work such as mask building and hit-finding-parameter tuning can be done via mouse operations. In the batch job module, different types of jobs (compressing, hit finding, peak2cxi conversion) can be submitted to the engine in manual or auto modes. The results can be used to refine parameters in the main GUI. The peak powder data generated from the main interface can be used in the geometry calibration module to optimize experimental geometry. All these modules work together to support preprocessing of SX data.

## 4. Availability   

The source code of *ClickX* is publicly available under a GPL license at GitHub (https://github.com/LiuLab-CSRC/ClickX). *ClickX* is compatible with both Python2 and Python3 environments. Dependencies include *NumPy*, *SciPy*, *mpi4py*, *scikit-image*, *pyqt5* (https://pypi.org/project/PyQt5/)and *pyqtgraph* (http://www.pyqtgraph.org/). A detailed introduction and usage instruction can be found at https://lixx11.github.io/ClickX.

## 5. Conclusion   

An SX preprocessing program, *ClickX*, has been developed with a user-friendly graphical interface for parameter tuning, batch job management and geometry calibration. *ClickX* is intended to support online/offline analysis for XFEL and synchrotron facilities that have the capability to conduct SX experiments. It has been thoroughly tested at PAL-XFEL and LCLS. *ClickX* is under active development and new features, such as support of time-resolved (Kupitz *et al.*, 2014 ▸; Tenboer *et al.*, 2014 ▸; Pande *et al.*, 2016 ▸; Nogly *et al.*, 2018 ▸) and two-color (Gorel *et al.*, 2017 ▸) serial crystallography experiments, will be integrated in future releases.

## Figures and Tables

**Figure 10:**
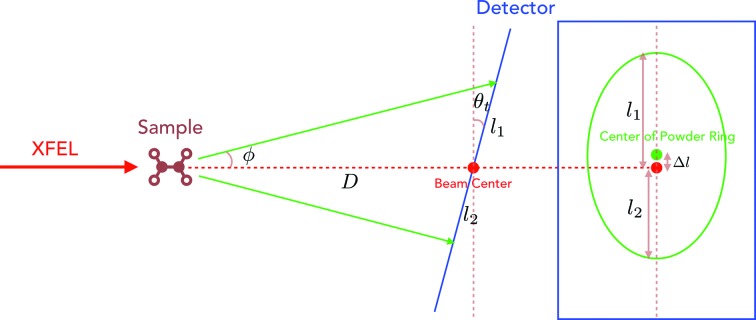
Schematic diagram of geometry calibration. The detector (blue) with small tilting angle is located downstream at a distance *D* from the powder sample. The resulting powder rings (green) exhibit an ellipse shape on the detector.

## Appendix A Geometry calibration

Fig. 10 ▸ shows a schematic diagram of the geometry calibration. Crystal samples are exposed to XFEL pulses, resulting in powder diffraction patterns with multiple rings. If the detector is tilted, the powder rings are not perfectly circular but elliptical with different centers.

For the powder ring at ϕ (, where θ is the scattering angle) on a detector with tilting angle , we can calculate the distances between the beam center and two endpoints of the long axis of the elliptical ring:

Then the distance between the beam center and the center of this elliptical ring is
